# Compulsory treatments in eating disorders: a systematic review and meta-analysis

**DOI:** 10.1007/s40519-020-01031-1

**Published:** 2020-10-24

**Authors:** Anna Rita Atti, Tomas Mastellari, Stefano Valente, Maurizio Speciani, Fabio Panariello, Diana De Ronchi

**Affiliations:** 1 Department of Biomedical and NeuroMotor Sciences, University of Bologna, Bologna, Italy; 2 Department of Mental Health, Local Health Authority of Bologna, Bologna, Italy

**Keywords:** Compulsory admission, Involuntary treatment, Involuntary hospitalization, Eating disorders, Anorexia nervosa, Bulimia nervosa, BMI, Meta-analysis

## Abstract

**Introduction:**

The aims of this systematic review and meta-analysis are to provide a summary of the current literature concerning compulsory treatments in patients with eating disorders (ED) and to understand whether compulsorily and involuntarily treated patients differ in terms of baseline characteristics and treatment outcomes.

**Methods:**

Relevant articles were identified following the PRISMA guidelines by searching the following terms: “treatment refusal”, “forced feeding”, “compulsory/coercive/involuntary/forced treatment/admission”, “eating disorders”, “feeding and eating disorders”, “anorexia nervosa”, “bulimia nervosa”. Research was restricted to articles concerning humans and published between 1975 and 2020 in English.

**Results:**

Out of 905 articles retrieved, nine were included for the analyses allowing the comparisons between 242 compulsorily and 738 voluntarily treated patients. Mean body mass index (BMI) was slightly lower in patients compelled to treatments. Mean illness duration, BMI at discharge and BMI variation showed no significant differences between the two groups. Average length of hospitalization was 3 weeks longer among compulsory-treated patients, but this did not result in a higher increase in BMI. No significant risk difference on mortality was estimated (three studies).

**Conclusions:**

Compulsory treatments are usually intended for patients having worse baseline conditions than voluntary ones. Those patients are unlikely to engage in treatments without being compelled but, after the treatments, albeit with longer hospitalisations, they do achieve similar outcomes. Therefore, we can conclude that forcing patients to treatment is a conceivable option.

**Level of evidence:**

Level I, systematic review and meta-analysis.

## Introduction

Eating disorders (ED) including among others, anorexia nervosa (AN) and bulimia nervosa (BN) are associated with severe morbidity and high mortality burden [1], have a serious impact on patient’s quality of life, and are responsible for increased healthcare utilization and costs [2].

Outpatient care is recommended by clinical guidelines [3, 4] for most of the patients affected by ED [5], including adolescents [6]; whereas for all persons with more severe clinical pictures, little or no differences between specialist inpatient care and active outpatient care (or a combination of both) have been demonstrated [7]. For patients with short illness duration and mild physical symptoms, primary treatment goals are restoring body weight and minimizing cognitive distortions. Conversely, according to a stage-matched intervention [8], in case of severe and enduring AN, treatment should be aimed to body weight stabilization, harm reduction, and improvements in psychosocial functioning [9, 10]. Similarly, in patients affected by BN, to break the binge/purge cycle, an outpatients’ setting is recommended, although hospitalization may occasionally be needed to force abstinence from binge/purge behaviors [11]. Besides symptoms severity [4], illness duration [12, 13], psychosocial functioning [14], and psychiatric comorbidities [15, 16] have been also claimed as relevant factors in the choice of the most appropriate treatment setting for both AN and BN [14] but, unfortunately, due to illness denial, engaging patients in out- or inpatients treatment can be challenging [17].

“Insight” in psychiatry encompasses the awareness of suffering from a mental illness, the understanding of the cause of such distress, and the acknowledgment of the need for treatment. Given these three assumptions, insight is necessarily a critical issue for patients’ treatment [18, 19]. For example, in studies carried out on patients affected by schizophrenia spectrum disorders, a clear relationship between low insight and poorer outcomes has been demonstrated [20, 21]; whereas for other psychiatric conditions, fewer data are available. To overcome the lack of insight, people affected by Severe Mental Illness (SMI) with impaired awareness of their health condition, when in need of care but refusing the therapies, might be legally committed to compulsory treatments. Persons affected by severe ED lack of a deep-rooted awareness of their body size and might not recognize neither the severity of their psychopathological condition nor the health-related risks of extreme fasting and purging behaviors [22]. Therefore, even if controversial [23], compulsory treatments might occasionally be necessary [24] and justifiable [25] being usually compelled to administer life-saving treatment [3, 26–29] and to prevent fatalities [30]. For example, in a large Danish sample of adult inpatients affected by AN, the use of involuntary measures was necessary in the 18% of cases [31]. The acceptance of an involuntary treatment, however, is debatable both from patients’ as well as from professionals’ side. Indeed, patients’ rights and autonomy are juxtaposed with professionals’ commitment to save lives and with their responsibility over patient’s health [23]. Unfortunately, the lack of a common legislation on this topic among different countries and the absence of shared protocols between care providers (even within the same country) make this controversial issue even more difficult to be handled.

The literature on compulsory treatments in ED is mainly based on ethical, philosophical and legal principles rather than on empirical data [24] and, due to ethical reasons, randomised controlled trials aimed to compare voluntary and compulsory treatments are missing. Many literature reviews have been carried out under different perspectives [19, 20, 26–30] and overall, there is a large agreement on the need of further qualitative and quantitative research to fill this gap; conversely, under a clinical point of view, results are inconclusive.

Discrepancies in literature findings are principally due to differences in study population composition (age, gender, diagnoses, and illness duration) and in kind of treatment (feeding or artificial nutrition, out- or inpatients). Further sources of discrepancies are compulsory treatments’ length (few days versus many weeks) and setting (psychiatric wards, general hospitals, residential homes) which depend on national legislations. Moreover, as the border between formal coercion and other forms of ‘strong persuasion’ in ED patients management is thin [33], treatments’ comparison becomes even more difficult. A further source of ambiguity in estimating treatments’ efficacy is related to the difficulties of defining appropriate outcomes: BMI restoration or binge–purging behaviors reduction, for example, is far from being appropriate outcomes to define an effective treatment. Efficacy should be alternatively evaluated based on psychopathology and functioning, which are also difficult to be quantitatively measured. The National Institute for Clinical Excellence (NICE) guidelines suggests that helping people to reach a healthy body weight for their age is a key goal which supports all the other psychological, physical and quality of life changes needed for improvement or recovery [3]. Unfortunately, as no agreement exists on the efficacy and usefulness of involuntary treatment for ED [23], in everyday clinical practice, professionals facing patients with severe ED stand alone in front of a difficult choice [35] being aware that compulsory hospitalizations demonstrated some benefits in the short term; whereas, in the long-term, have been proven to undermine the psychotherapeutic relationship and increase drop-out rates [34].

The aims of the present systematic review and meta-analysis are multiple. First, we sought to provide an update of the literature on this topic because a few studies [31, 36, 37] have been published since the last two reviews published in 2014 [24, 38] were carried out. The second aim is to compare some of the features of patients voluntarily and compulsorily admitted identifying who is more likely to be compelled to treatments. Third, we were interested in estimating the extent to which voluntary and compulsory treatments yield different outcomes.

## Materials and methods

### Search strategy

Relevant articles were identified following the preferred reporting items for systematic reviews and meta-analyses (PRISMA) guidelines [39]. An extensive electronic database literature search was conducted in PubMed, PsycINFO, Scopus and Medline. These databases offer optimal coverage of relevant literature in the medical field (https://handbook-5-1.cochrane.org/chapter_6/6_searching_for_studies.htm).

The following terms were used: “treatment refusal”, “forced feeding”, “compulsory/coercive/involuntary/forced treatment/admission”, “eating disorders”, “feeding and eating disorders”, “anorexia nervosa”, “bulimia nervosa”. Research was restricted to articles published in English and concerning humans between 1st January 1975 and 10th July 2020. Additional articles were also found via hand searches of the reference lists of all retrieved articles.

### Selection procedure

Two investigators (MS and TM) independently screened all relevant articles according to the following inclusion criteria: (1) presence of a study design based on a two-group comparison (compulsory versus voluntary); (2) presence of a description of socio-demographic and clinical characteristics of enrolled patients (age, gender, diagnosis, duration of illness and/or body mass index progression); and (3) presence of information on the hospitalization (length of stay, type). We excluded articles with different study design, reviews, case reports, commentaries and legal or ethical discussions. In case of disagreement on including or excluding articles between the two investigators, a senior author was involved for final decision (ARA).

### Quality evaluation

The quality of included studies was assessed by the Newcastle Ottawa scale [40]. According to this scale, largely used for evaluating the value of non-randomized studies in meta-analyses, three factors need to be considered to score the quality of included studies. First, selection of the exposed and of the non-exposed cohort and ascertainment of exposure; second, comparability, assessed on the basis of study design and analysis, and whether any confounding variables were adjusted for; and third, the outcome which is evaluated on the basis of the follow-up period and cohort retention. The quality of the studies (good, fair and poor) is then rated by awarding stars in each domain: a “good” quality score requires 3 or 4 stars in selection, 1 or 2 stars in comparability, and 2 or 3 stars in outcomes; a “fair” quality score requires 2 stars in selection, 1 or 2 stars in comparability, and 2 or 3 stars in outcomes and, finally, a “poor” quality score reflects 0 or 1 star(s) in selection, or 0 stars in comparability, or 0 or 1 star(s) in outcomes.

### Statistical analyses

This meta-analysis was conducted on Review Manager 5.3 (Cochrane Collaboration software). Data are presented as mean differences or risk differences (RD), considering 95% confidence intervals (CI). To provide a valid estimation of the pooled effect of included articles Random Effect models were used. Forest Plots were then created for graphical presentations of collected data. Based on the available information, the following outcomes were considered: length of stay, illness duration, BMI at admission, BMI at discharge, BMI variation, and mortality.

Heterogeneity, the variation in study outcomes between studies was tested by a Chi-square test and a *P* value lower than 0.05 excluded the presence of statistically significant heterogeneity. The heterogeneity coefficient *I*^2^ indicates that heterogeneity among studies. *I*^2^ is null or might not be important when ranging between 0 and 40%, moderate when ranging between 30 and 60%, substantial when ranging between 50 and 90% and considerable when between 75 and 100% (https://handbook.cochrane.org/chapter_9/9_5_2_identifying_and_measuring_heterogeneity.htm). Issue of heterogeneity in the present meta-analysis have been addressed by sensitivity analyses.

## Results

**Fig. 1:**
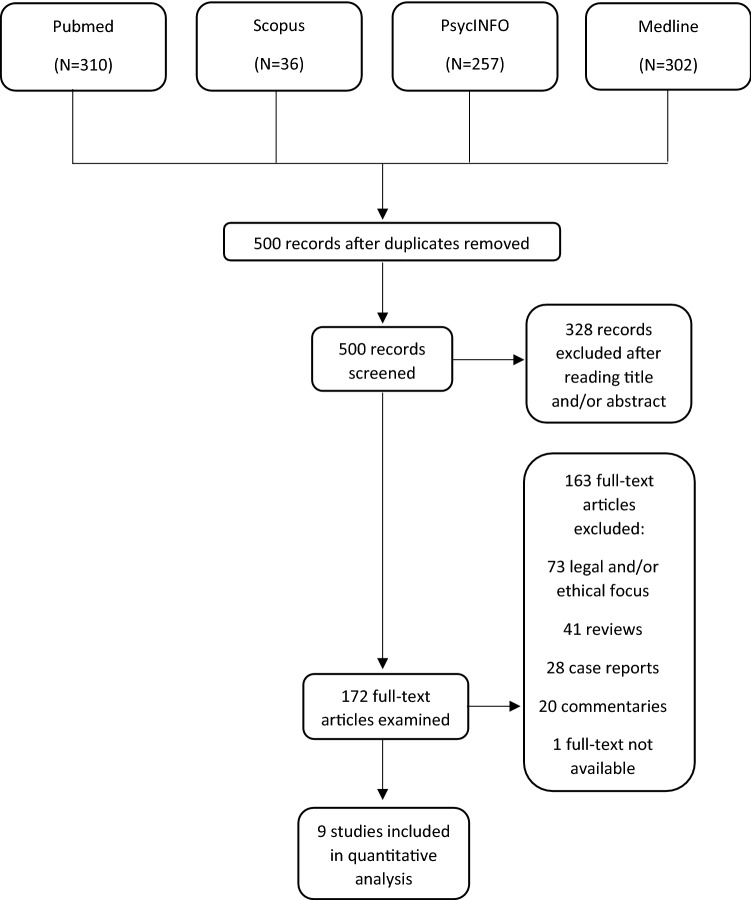
Flowchart representing the study selection procedure

Figure 1 illustrates in detail the procedure of articles’ selection. Out of 905 articles retrieved, 405 duplicates were removed and 500 were left. After reading title and or abstract, 328 were further excluded and 172 were extensively examined. After reading the full text, 73 articles with a legal or ethical perspective, 41 reviews, 28 case reports, 20 commentaries, and one full text not available were eliminated leaving nine articles available for the systematic revision and the meta-analysis.

Table 1 reports a detailed description of the nine studies included in the quantitative analysis. Overall, 242 compulsory-treated patients are compared to 738 voluntary patients. The large majority are female. Mean age ranges between 16 and 37.6 years and illness duration between 1.9 and 18.3 years. All articles have a naturalistic retrospective study design and are carried out in Units specialized in the treatment of ED situated in the United Kingdom [37, 41–43], Australia [44–46], the United States [47] and Norway [36]. All studies but one [46] are embedded in a single clinical setting and enrolled patients over the years (between two and twelve years). Some of them collected information through medical record and national registry but only four studies [37, 41–43] reported data on mortality. Even if AN was the most common clinical diagnosis among patients treated compulsorily, there were also cases of treatments compelled to patients affected by BN or eating disorders not otherwise specified (EDNOS).

**Table 1:** Longitudinal retrospective studies investigating differences between compulsorily and voluntarily admitted patients

Author/year	Patients	Age, years	Female	Diagnoses	Illness duration, years	Admission BMI, kg/m^2^	Discharge BMI, kg/m^2^	∆BMI, %	Length of stay, weeks	Follow up, years
	(*n*)	(Mean ± SD)	(%)		(Mean ± SD)	(Mean ± SD)	(Mean ± SD)		(Mean ± SD)	(Mean ± SD)
Ramsay et al*.* [42]	*C* = 81	26.2 ± 9.5	93.8%	AN; BN; EDNOS^‡^	8.2 ± 6.1	14.2 ± 2.7	18.7 ± 2.3	31.7%	16.1 ± 12.9	5.7 ± 3.3
Ward et al*.* [37]	*V* = 81	25.4 ± 7.7	97.5%	AN; BN; EDNOS^‡^	7.6 ± 6.4	14.3 ± 2.4	18.5 ± 2.0	29.4%	12.6 ± 7.6	5.7 ± 3.3
Watson et al*.* [47]	*C* = 66	F 24.7 ± 7.1 M 28.0 ± 11.4	90.9%	AN: F 41.7%; M 50% BN: F 26.7%; M 33.3% EDNOS: F 31.7%; M 16.7%^a^	1.9 ± 1.5	17.4 ± 4.7	20.5 ± 3.8	17.8%	8.2 ± 6.6	Not reported
	*V* = 331	F 24.9 ± 8.7 M 23.9 ± 9.2	87.9%	AN: F 43.3%; M 60% BN: F 26.1%; M 17.5% EDNOS: F 30.6%; M 22.5%^a^	1.6 ± 1.6	18.4 ± 4.7	20.7 ± 3.6	12.5%	5.8 ± 5.1	Not reported
Ayton et al*.* [41]	*C* = 16	16.2 ± 1	Not reported	AN-R: 56.3%; AN-BP: 6.3%; EDNOS: 37.5%^a^	3.8 ± 2.1	16.6 ± 2.6	19.6 ± 1.5	18.1%	55.6	1
	*V* = 34	16.2 ± 1.3	Not reported	AN-R: 67.6%; AN-BP: 14.7%; EDNOS: 17.6%^a^	1.9 ± 1.5	14.2 ± 1.9	18.5 ± 1.6	30.3%	32	1
Serfaty et al*.* [43]	*C* = 7	37.6 ± 14.2	100%	AN-BP: 14.3%; AN-R: 85.7%^a^	16.3 ± 10.7	11.8 ± 2.3	17.1 ± 2.7	44.9%	Not reported	0.9 ± 0.7
	*V* = 4	25 ± 6.5	100%	AN-BP: 50%; AN-R: 50% ^a^	9.0 ± 6.8	12.0 ± 1.2	16.6 ± 0.6	38.3%	Not reported	1.1 ± 0.6
Griffiths et al*.* [46]	*C* = 15 *V* = 73	23.76 ± 7.87 20.7 ± 7.4	100% Not reported	AN^c^ AN^c^	8.3 Not reported	13.4 ± 1.8 14.3 ± 2.2	18.1 ± 2.1 17.2 ± 2.9	34.6% 20.3%	14.9 ± 10.9 8.8 ± 6.0	1 Not reported
Carney et al*.* [44]	*C* = 26^d^	24.5 ± 8.57	96.2%	AN-R: 76.9%; AN-BP: 23.1%^a^	8.1 ± 7.6	13.2 ± 1.7	14.9 ± 1.4	12.7%	7.4 ± 6.7	Not reported
Carney et al*.* [45]	*V* = 70^d^	24.84 ± 7.46	95%	AN-R: 67.1%; AN-BP: 32.9^a^	6.3 ± 6.5	14.0 ± 1.8	15.4 ± 2.3	9.6%	6.8 ± 7.6	Not reported
Halvorsen et al*.* [36]	*C* = 31^d^ *V* = 145^d^	21.1 ± 5.7 22.4 ± 8.6	96% 96%	AN^b^ AN^b^	5.8 ± 4.2 7.1 ± 7.6	14.1 ± 2.0 15.0 ± 1.7	16.6 ± 2.0 17.8 ± 1.7	17.7% 18.7%	34.2 ± 21.9 23.6 ± 15.5	Not reported Not reported

*n* number of patients/number of previous admissions, *C* compulsory patients group, *V* voluntary patients group, *F* female, *M* male, *ICD* International Classification of Diseases, *DSM* diagnostic and statistical manual of mental disorders, *AN* anorexia nervosa, *AN-R* restrictive anorexia nervosa, *AN-BP* binge–purging anorexia nervosa, *EDNOS* not otherwise specified eating disorders, *BMI* body mass index, *∆ BMI* difference between discharge and admission BMIs

^a^DSM-4 diagnostic criteria

^b^Proportion and criteria not reported

^c^DSM-4 and ICD-10

^¶^Number of admissions

In Table 2, the occurrence of prior hospitalizations, the extent of psychiatric comorbidities as well as mortality rates are described. All studies but two [36, 43] reported information on prior hospitalizations that were more frequent in the compulsory group compared to the voluntary one. Apart from two studies [36, 43] which missed to report information about psychiatric comorbidities, in the remaining studies, the occurrence of depression, substance abuse and self-harm was higher in the compulsory- than in the voluntary-treated patients. Unfortunately, the wide heterogeneity of the type of information provided about comorbidity precluded us the possibility of meta-analyzing the data.

**Table 2:** Longitudinal retrospective studies investigating previous admissions, mortality and/or psychiatric comorbidities in compulsorily and voluntarily admitted patients

Author/year	Patients	Previous admissions	Mortality	Psychiatric comorbidities	Study quality (Ottawa Score)			
	(*n*)	(mean ± SD) or (%)	(%)	(%)	Selection	Comparability	Outcome	Total
Ramsay et al*.* [42]	*C* = 81	3.3 ± 3.2	12.3	Self-harm 59.3%	4	2	2	8
Ward et al*.* [37]	*V* = 81	1.8 ± 2.2	12.3	Self-harm 33.3%	3	2	3	8
Watson et al. [47]	*C* = 66	3.0 ± 7.4	–	Depression 45% (F); 66.7% (M) Substance abuse 30% (F); 16.7% (M)	3	1	1	6
	*V* = 331	1.4 ± 3.3	–	Depression 41.9% (F); 40% (M) Substance abuse 25.1% (F); 12.5% (M)				
Ayton et al*.* [41]	*C* = 16	87.5% of *C* ≥ 1	0	Depression 96.6%; OCD 12.5%; ASD 18.6%; Self-harm 75%	3	2	1	6
	*V* = 34	29.4% of *V* ≥ 1	6.9	Depression 58.8%;OCD 14.7; ASD 11.8%; Self-harm 11.8%				
Serfaty et al*.* [43]	*C* = 7	–	0	–	2	0	1	3
	*V* = 4	–	0	–				
Griffiths et al*.* [46]	*C* = 15	Inpatients 40%; Outpatients 6.7%; Both 40%	–	60% (affective disorder 26.7%; OCD 6.7%; schizophrenia 6.7%; personality disorder 20%; attempted suicide 20%; substance abuse 13.3%)	4	1	1	6
	*V* = 73	Inpatients 42%; Outpatients 40%	–	–				
Carney et al*.* [44]	*C* = 26¶	3.9	–	85%	4	1	1	6
Carney et al*.* [45]	*V* = 70	1.7		70%	4	1	3	8
Halvorsen et al*.* [36]	*C* = 31 *V* = 145¶				3	2	3	8

*n* number of patients/number of previous admissions, *C* compulsory patients group, *V* voluntary patients group, *M* males, *F* females, *OCD* obsessive compulsive disorder, *ASD* autistic spectrum disorder

^¶^Number of admissions

### Illness duration

**Fig. 2:**
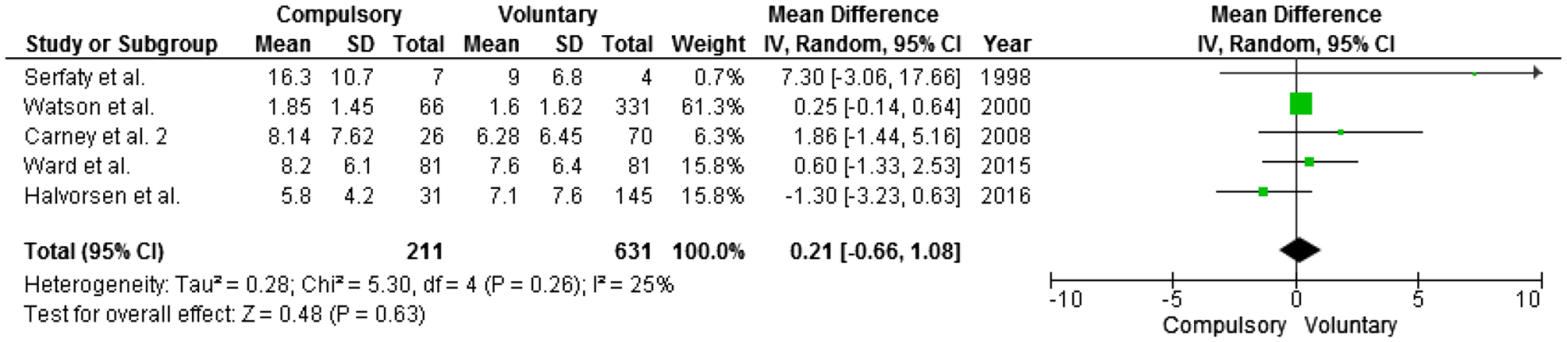
Illness duration. Mean difference is computed as duration in compulsory treatment minus duration in voluntary treatment; thus, negative mean differences suggest that the compulsory group has a longer mean illness duration than the voluntary one

Information on illness duration (before treatment) was reported in 5 articles. Illness duration was longer in the compulsorily treated groups in three articles [37, 43, 45], was shorter in another article [36] and was similar in the last one [47]. Overall, mean illness duration was not statistically significantly different between the compulsory and voluntary group (Fig. 2).

### BMI

**Fig. 3:**
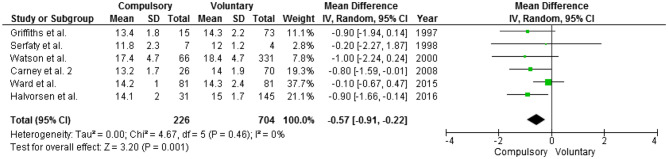
BMI at admission. Mean difference is computed as BMI at admission in compulsory minus BMI at admission in voluntary; thus, negative mean differences suggest that the compulsory group has mean lower BMI than voluntary one

Data on mean BMI at admission were recorded in six articles. All studies but one [47] concerned patients with mean BMI equal or lower than 15 kg/m^2^. The lowest mean BMI (11.8 ± 2.3 kg/m^2^) was reported in the compulsory group by Serfaty and colleagues in a small study published in 1998 [43]. Although only two studies [36, 45] reported a statistically significant difference in mean BMI at admission between groups, overall the compulsory-treated patients (*N* = 226) had a slightly lower mean BMI compared to the voluntary ones (*N* = 704) with an estimated mean difference equal to 0.57 kg/m^2^ (confidence interval: − 0.22 to − 0.91) (Fig. 3). When only studies involving patients affected by AN were considered, results were confirmed with a mean BMI difference between the compulsory and the voluntary groups equal to −0.84 kg/m^2^ (confidence interval: − 1.30 to − 0.37) (data not shown).

**Fig. 4:**
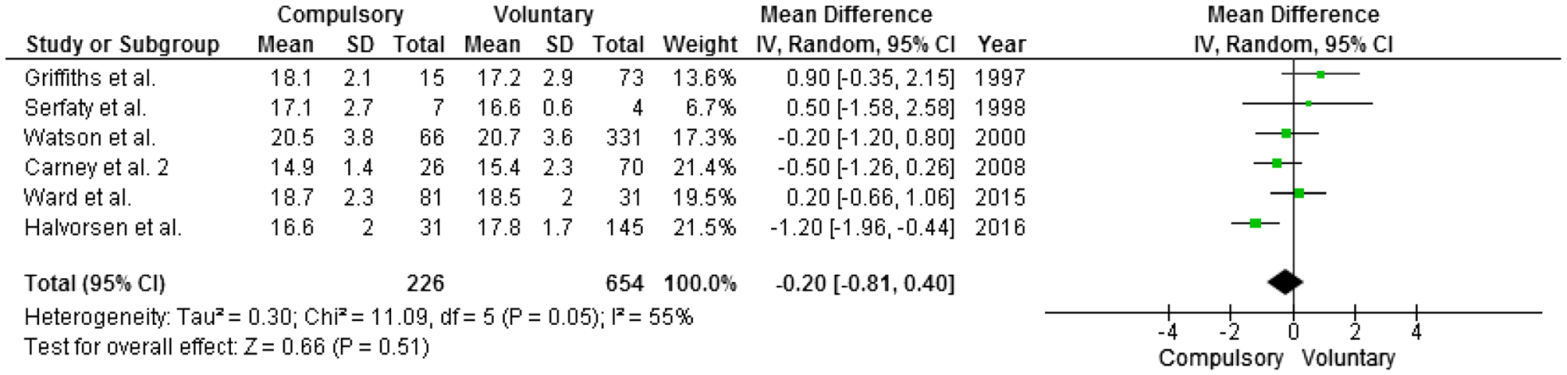
BMI at discharge. Mean difference is computed as BMI at discharge in the compulsory group minus BMI in the voluntary group; thus, negative mean differences suggest that the compulsory group has mean lower BMI than the voluntary one

No differences were detectable on BMI at discharge in the six articles reporting this information (Fig. 4). When only articles on patients affected by AN were considered, differences were similar and not statistically significant. Noticeably, regardless of type of treatment, in four [36, 43, 45, 46] out of six studies, BMI at discharge remained below 18.5 kg/m^2^.

**Fig. 5:**
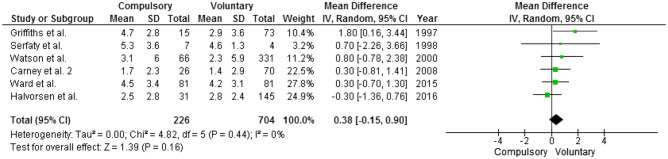
BMI variance (post- minus pre-treatment BMI). Mean difference is computed as post-treatment BMI in the compulsory group minus post-treatment BMI in the voluntary group; thus, positive mean differences suggest that the compulsory group on average has a higher post-treatment BMI than the voluntary one

In all studies reporting both BMI at admission and at discharge, BMI variation was computed as the difference between post- and pre-treatment BMI. The overall estimated variation was in favor of the compulsory group that gained, on average, 0.38 kg/m^2^ more than the voluntary group but such minimal difference was not statistically significant (confidence interval: − 15.00 to + 0.90) (Fig. 5). When only studies that enrolled patients with AN were considered, results were unchanged. Since BMI changes during treatment might be influenced by initial BMI, percentages of changes were also computed. On average, the compulsory-treated patients gained the between 12.9 and 36.4% of admission BMI; whereas, voluntary-treated patients gained between 10 and 38.3% but again such difference was not statistically significant (mean BMI difference 3.8%; confidence interval: − 0.52 to + 7.02). Neither results were influenced by the inclusion of the studies including only patients affected by AN.

### Hospitalization

**Fig. 6:**
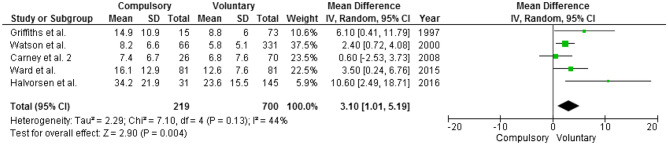
Length of hospitalization. Mean difference is computed as length of hospitalization in the compulsory group minus length of hospitalization in the voluntary group; thus, positive mean differences suggest that the compulsory group has on average a longer hospitalization than the voluntary one

Information on length of hospitalization were available in 5 articles [36, 37, 45–47]. Length of stay varied widely (range 5.8–34 weeks) being on average three weeks longer among compulsory-treated patients than among voluntary ones (Fig. 6).

### Mortality

**Fig. 7:**
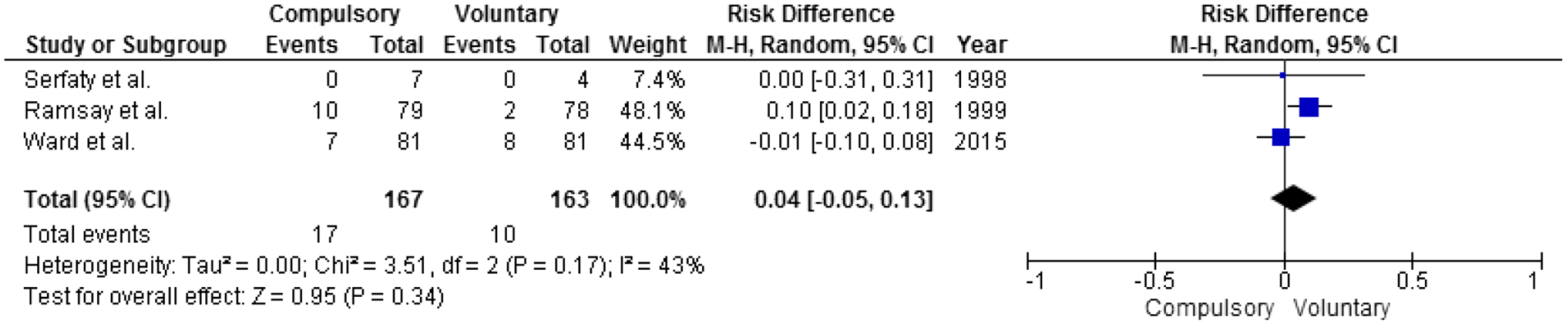
Mortality

The three studies [37, 41–43] reporting data on mortality were characterized by some degree of variability in follow-up length (range 0.9–5.7 years); however, no risk difference emerged between those treated compulsorily (*N* = 167) and voluntarily (*N* = 163) (Fig. 7).

### Heterogeneity and sensitivity analyses

Heterogeneity was not relevant (*I*^2^ < 40%) neither statistically significant (*p* > 0.05) in most of the analyses (details are reported below each Forest Plot). Regarding BMI at discharge (Fig. 4), heterogeneity was moderate (*I*^2^ = 55%) and approached statistical significance (*p* = 0.05). To overcome this limitation and provide a more exhaustive description of the available studies, we further implemented the sensitivity analyses described below.

All the previous analyses were additionally performed after the inclusion of the unique article that enrolled patients below legal age [41]. In this study , 16 compulsorily treated and 34 voluntarily treated adolescents with a restrictive AN and comorbid depression were followed up for one year. The inclusion in the meta-analyses of this study did not changed the overall results. Since median illness duration was 7 ± 6.3 years, all the analyses were stratified accordingly: for studies with illness duration shorter than 7 years [36, 45, 47] and for studies with illness duration equal or longer than 7 years [37, 43, 46], results were similar. Apart from one study [43] that did not report information on length of hospitalization, these data were available in the other eight studies [36, 37, 41, 42, 44–47]. As median length of hospitalization was 13.8 weeks, all the analyses were stratified accordingly. Results of studies with shorter [37, 44, 45, 47] and longer [36, 41, 42, 46] length of hospitalization were unchanged.

Applying the Newcastle Ottawa Scale, the quality of four [36, 37, 42, 45] studies was judged as very good (for details see Table 2). A sensitivity analysis run only with those studies demonstrated that BMI at admission was slightly lower in the 138 compulsory hospitalized patients compared to the 296 voluntary admissions with a mean difference equal to minus 0.62 (− 1.12 to − 0.12). No differences were detectable between the two groups at the end of the treatment. Further additional analyses run only on studies which reported information on psychiatric comorbidity in both the voluntarily and the compulsorily treated groups gave similar results.

## Discussion

The present systematic review of the literature and meta-analysis confirm the paucity of studies on compulsory treatments in ED and highlight the need of further research in this neglect area. The nine articles used for the present quantitative synthesis have a naturalistic study design and are mainly based on retrospective data collection; nevertheless, they provide a body of evidences on 242 patients affected by ED treated against their will. Findings of our meta-analysis can be summarized as follows:

(i) Compulsory treatments are compelled to patients with different clinical diagnoses, illness duration, and psychiatric comorbidity.
(ii) BMI after treatments is similar in the compulsory and in the voluntary groups. Sometimes treatments’ end occurs when body weight is not yet fully restored.
(iii) Even if average length of hospitalization is three weeks longer among compulsory-treated patients, this does not result in a superior increase in BMI.
(iv) No significant Risk Difference on mortality exists between voluntarily and compulsorily treated patients (three studies available).

Since starvation can be a life-threating condition, compulsory treatments are forecast to be more likely in patients with lower BMI. Findings from our meta-analysis confirmed that overall BMI was statistically significant lower in the compulsory group, but the difference (0.57 kg/m^2^) was not clinically relevant. There is a general agreement upon the fact that BMI is far from being a satisfactory measure of illness severity, but, unfortunately, the current available literature did not provide alternative information as only three [41, 46, 47] studies reported different psychopathological measures that anyway were not comparable. Illness duration is assumed to be a further possible markers of illness severity. Indeed, illness duration was 1.7 years longer in the compulsory-treated group, but such difference was not statistically significant. Since the co-occurrence of depression, substance abuse and self-harm with ED could overturn the balance in favors of choosing a compulsory treatment, we focused our attention also on psychiatric comorbidity but the studies providing such information were few and too heterogeneous for a meta-analysis.

BMI at discharge and BMI difference after treatment between the compulsory and the voluntary groups were similar regardless of baseline BMI in all ED patients as well as in AN patient. Although BMI is not the most satisfactory measure to judge treatment efficacy in ED [48, 49], such result demonstrates that compulsory treatments are neither superior nor inferior to voluntary ones in influencing weight gain. It is noteworthy that in some studies, BMI at discharge did not exceed 18.5 which is the WHO threshold identifying underweight [50]. According to a clinical perspective, this suggests that compulsory treatments per se are meaningful and need to be integrated in a long-lasting treatment program. Interestingly, the average length of hospitalization for compulsory-treated patients was longer (and presumably related costs were higher) without having a significant impact on BMI changes.

The main unresolved issue in judging the effectiveness of compulsory treatment in ED patients is that we do not know what would have happened to the patients who underwent compulsory treatment if they had not been compelled to such treatment. Unfortunately, this precludes the possibility of drawing any definitive conclusions; however, as the outcomes between compulsory and voluntary-treated patients did not differ, we can assume that there are no beneficial neither detrimental effects in the two types of treatment. Since patients who have been compelled to treatment appeared generally worse at baseline than the voluntary group, and given that they are highly unlikely to engage in treatment without being compelled to do so, whilst they achieve similar outcomes, albeit with longer length of stays, we can conclude that obliging the patients to the treatment does not undermine the effectiveness of the treatment itself and not forcing them does not reduce the probability of a favorable outcome.

Furthermore, when patients compulsorily and voluntarily treated were compared, mortality was neither inferior nor superior. It is well acknowledged that AN has the highest mortality rate of any psychiatric illness and that mortality risks factors include illness chronicity, critically low body weight, and bingeing and purging behaviors. Given the delusional beliefs concerning patients’ body image and the impaired cognitive performance caused by starvation, an accurate evaluation of individual’s risk is mandatory and every effort of treating life-threatening cases must be implemented [25].

Unfortunately, all the publications on patient’s (and family’s) perspective on this topic have a qualitative design that prevented the possibility to include those study in this meta-analysis. Conversely, addressing patient’s point of view would have been relevant: for example, patients reported that a trusting relationship with health professionals could even prevent the admission to be perceived as coercive [51]; furthermore, the degree of satisfaction after discharge was similar between voluntary and involuntary admissions [52]. Guarda et al. [53] found that nearly half of the patients who denied the need for treatment acknowledged it in just two weeks of inpatient care. In addition, Tan et al. [51] reported that while there were some differences in patients’ and parents’ perception of what constitutes capacity to choose for their own health, they all agreed that it would not be right to allow someone to die as a result of respecting their refusal of treatment.

Some limitations of the present meta-analysis deserve a comment. It should be noted that compulsory treatments are not an alternative form of therapy but rather a choice of care responding to different conditions: lack of insight, lack of compliance to therapy, psychopathological and medical severity. Therefore, every comparison between compulsory and involuntary treatments should be considered cautiously. As predictable, psychiatric comorbidities were more frequent in compulsory-treated patients, who also had a slightly lower BMI. Unfortunately, the small number of studies included in the meta-analysis and the reduced sample size of most of them prevented us from the possibility of running moderator analyses. Given the non-randomized nature of the studies included in this meta-analysis, pre-treatment differences between groups are the most relevant limitations to compulsory versus voluntary treatment comparisons. However, we used percentage of BMI changes to reduce the bias related to different baseline conditions.

To conclude, the available literature does not offer to the clinicians’ useful information for compelling compulsory treatment or not and for predicting who will get benefits and who will not. However, as full recovery is possible even in severe ED cases, compulsory treatments should not be prohibited and might be justifiable and necessary [25]. As already happening for other psychiatric disorders, the preventive setting up of a joint crisis plan in which patient’s treatment preferences for any future emergency are shared with the clinicians could be a collaborative strategy that further improves the therapeutic relationship [54]. To reduce patient’s perception of coercion, the implementation of an outpatient- rather than an inpatient-compulsory treatment could be helpful. Similarly, the type of ward (a nutritional or internal medicine ward rather than a psychiatric locked ward) in which the compulsory treatment takes place could be relevant to improve patients’ acceptability and fight stigma.

### What is already known on this subject?

Compulsory treatments might be life-saving and can provide some benefits in the short term; whereas, in the long term, could undermine the therapeutic relationship.

### What this study adds?

This study updates the literature on a neglected area of research suggesting that forcing ED patients to treatment is a conceivable option.
